# Carotid intimal-media thickness as a surrogate for cardiovascular disease events in trials of HMG-CoA reductase inhibitors

**DOI:** 10.1186/1468-6708-6-3

**Published:** 2005-03-10

**Authors:** Mark A Espeland, Daniel H O'Leary, James G Terry, Timothy Morgan, Greg Evans, Harald Mudra

**Affiliations:** 1 Department of Public Health Sciences, Wake Forest University School of Medicine, Winston-Salem, North Carolina, USA; 2 Department of Radiology, Tufts University School of Medicine, Boston, Massachusetts, USA; 3 Department of Internal Medicine, Wake Forest University School of Medicine, Winston-Salem, North Carolina, USA; 4 Medical Department, Krankenhaus München Neuperlach, Germany

**Keywords:** Arteriosclerosis, carotid arteries, drugs, meta-analysis, statistics, ultrasonics

## Abstract

**Background:**

Surrogate measures for cardiovascular disease events have the potential to increase greatly the efficiency of clinical trials. A leading candidate for such a surrogate is the progression of intima-media thickness (IMT) of the carotid artery; much experience has been gained with this endpoint in trials of HMG-CoA reductase inhibitors (statins).

**Methods and Results:**

We examine two separate systems of criteria that have been proposed to define surrogate endpoints, based on clinical and statistical arguments. We use published results and a formal meta-analysis to evaluate whether progression of carotid IMT meets these criteria for HMG-CoA reductase inhibitors (statins).

IMT meets clinical-based criteria to serve as a surrogate endpoint for cardiovascular events in statin trials, based on relative efficiency, linkage to endpoints, and congruency of effects. Results from a meta-analysis and post-trial follow-up from a single published study suggest that IMT meets established statistical criteria by accounting for intervention effects in regression models.

**Conclusion:**

Carotid IMT progression meets accepted definitions of a surrogate for cardiovascular disease endpoints in statin trials. This does not, however, establish that it may serve universally as a surrogate marker in trials of other agents.

## 

Atherosclerosis is a generalized disease that causes lesions in large- and medium-sized elastic and muscular arteries. As lesions progress, arterial walls are remodeled, a process through which the size of the arterial lumen is preserved. Because of this, the disease is clinically asymptomatic during its earlier stages and may go unnoticed for decades as the risk for its clinical manifestation as acute vascular disease grows [1,2]. Epidemiological studies and intervention trials based on the incidence of acute vascular disease endpoints require years of follow-up, the participation of large populations, or both. As a consequence, such studies consume considerable time and financial resources [3].

The use of surrogate markers for atherosclerosis extent and progression is widespread. Currently, the most established of these is based on carotid intima-media thickness (IMT) as measured by B-mode ultrasound. It is a natural extension to consider these measures as surrogate markers for cardiovascular disease clinical endpoints [4,5]. If this extension is valid, the time, expense, and participant burden in understanding and developing treatments to reduce the risk of clinical endpoints can be reduced. To be rigorous, this definition must be based on accepted definitions and/or set of criteria for surrogacy. This document examines the evidence that carotid IMT, a marker for atherosclerosis, meets two prominent set of criteria for defining surrogate outcomes.

## Defintions of surrogate markers

Both clinical and statistical criteria for surrogacy have been proposed.

### Clinical Criteria for Surrogacy

Boissel, et al. lay out criteria that markers must meet to be considered as valid surrogates for clinical endpoints [6]. We group these into three domains.

**B1: **(Efficiency) The surrogate marker should be relatively easy to evaluate, preferably by non-invasive means, and more readily available than the gold standard. The time course of the surrogate should precede that of the endpoints so that disease and/or disease progression may be established more quickly via the surrogate. Clinical trials based on surrogates should require fewer resources, less participant burden, and a shorter time frame.

**B2: **(Linkage) The quantitative and qualitative relationship between the surrogate marker and the clinical endpoint should be established based on epidemiological and clinical studies. The nature of this relationship may be understood in terms of its pathophysiology or in terms of an expression of joint risk.

**B3: **(Congruency) The surrogate should produce parallel estimates of risk and benefit as endpoints. Individuals with and without vascular disease should exhibit differences in surrogate marker measurements. In intervention studies, anticipated clinical benefits should be deducible from the observed changes in the surrogate marker.

### Statistical Criteria for Surrogacy

Prentice views surrogacy as a statistical property and defines it with mathematical expressions [7,8]. Four criteria are required for S to serve as a surrogate for endpoint T with respect to intervention Z.

**P1: **The intervention should affect the distribution of T.

**P2: **The intervention should affect the distribution of S.

**P3: **The distribution of T should be dependent on S.

**P4: **Endpoint T should be conditionally independent of Z given S, i.e. S should fully account for the impact of Z on T.

This definition may be specific to a particular setting and cohort; a marker may meet the criteria for surrogacy for one intervention, but fail criteria for others. The criteria for surrogacy are based on explicit models, and may also be dependent on covariates and additional explanatory factors being collected and incorporated into these models.

### Establishing Surrogacy

These clinical and statistical definitions require different approaches to establish surrogacy, neither of which is clear-cut. To meet the criteria outlined by Boissel, et al. [6], experience and data from clinical trials are required to demonstrate efficiency and congruence, and data from bench and cohort studies are required to establish plausible linkage. Arguments for surrogacy address whether these data are sufficiently compelling. To meet the criteria outlined by Prentice [7], decisions must be made on the parametric model describing the relationship between intervention and outcomes. The plausibility for surrogacy is argued by the ability of the surrogate marker, once incorporated in this model, to account (induce conditional independence) for this relationship using experimental data (and by P1 is limited to interventions that affect outcomes). Since statistical relationships cannot be established with certainty, arguments are required that the empirical evidence for conditional independence provided by data are sufficiently compelling to adopt the hypothesis of conditional independence required by the Prentice criteria.

## B-mode ultrasound imt

B-mode ultrasound imaging technology has evolved to the extent that the walls of superficial arteries can be imaged non-invasively, in real-time, and with high resolution. Unlike angiography or 'luminology', ultrasound imaging can visualize the arterial wall at every stage of atherosclerosis, from 'normal' arterial wall to complete arterial occlusion. Arterial wall thickness can therefore be measured as a continuous variable from childhood to old age, in patients and healthy controls [9]. Studies that have evaluated the origin of the lumen-intima and the media-adventitia ultrasound interfaces in relation to carotid and femoral far-wall arterial histology have demonstrated that the distance between these interfaces reflects the intima-media complex. Consequently, this distance is referred to as IMT [10,11]. IMT has been widely used in both observational studies and intervention studies.

## Surrogacy of carotid imt with respect to statins

We are interested in examining the potential of carotid IMT to serve as a surrogate marker for cardiovascular events, in particular cardiovascular mortality, myocardial infarctions, and clinical stroke. The clinical and statistical arguments for surrogacy are contextual, i.e. are based on specific relationships and mechanisms. It is unreasonable to make open-ended claims that a carotid IMT is a surrogate for these endpoints for all interventions and all cohorts, a point that has not been emphasized sufficiently.

Our specific focus is to examine surrogacy in clinical trials of HMG-CoA reductase inhibitors (statins). Empirical evidence is largely drawn from statin clinical trials conducted on cohorts of adults at elevated risk for cardiovascular endpoints. Our choice of statins is based, in part, on the many published trials available for these agents. We acknowledge that it is quite possible that IMT may be a valid surrogate for cardiovascular events with respect to statins (i.e. accounting for the effects of these agents on cardiovascular events), but may not be a valid surrogate for other agents (e.g. diuretics or postmenopausal hormone therapy) or endpoints. We used Medline searches to identify seven placebo-controlled clinical trials of statins that report both IMT outcomes and cardiovascular events (see Table 1): the Asympotomatic Carotid Artery Progression Study (ACAPS), the Kuopio Atherosclerosis Prevention Study (KAPS), the Pravastatin, Lipids, and Atherosclerosis in the Carotid Arteries Study (PLAC-2), the Carotid Atherosclerosis Italian Ultrasound Study (CAIUS), the Regression Growth Evaluation Statin Study (REGRESS), the Beta-Blocker Cholesterol Lowering Asymptomatic Plaque Study (BCAPS), and the Fukuoka Atherosclerosis Trial (FAST) [12-18].

**Table 1:** Clinical trials involving HMG-CoA reductase inhibitors and reporting both carotid IMT and cardiovascular event outcomes.

Clinical Trial (N*)	Statin	Relative Impact on IMT Progression of Primary Outcome (mm/yr): Mean [95% CI] (reported p-value)	Relative Impact on Reported Cardiovascular Endpoints: Odds Ratio [95% CI]	
			
			Abstracted CVD Event	Odds Ratio
ACAPS^(25) ^(N = 919)	Lovastatin	-0.015 [-0.023, -0.007] (p = 0.001)	CVD Death, MI, Stroke	0.34 [0.12, 0.69]
KAPS^(26) ^(N = 447)	Pravastatin	-0.014 [-0.022, -0.006] (p = 0.005)	CVD Death, MI, Stroke	0.57 [0.22, 1.47]
PLAC-II^(47) ^(N = 151)	Pravastatin	-0.009 [-0.031, 0.013] (p = 0.44)	Clinical Coronary Events	0.37 [0.11, 1.24]
CAIUS^(48) ^(N = 305)	Pravastatin	-0.014 [-0.021, -0.005] (p = 0.0007)	CVD Death, MI	1.02 [0.14, 7.33]
REGRESS^(28) ^(N = 255)	Pravastatin	-0.030 [-0.056, -0.004] (p = 0.002)	Clinical Events	0.51 [0.24, 1.07]
BCAPS^(49) ^(N = 793)	Fluvastatin	-0.008 [-0.013, -0.003] (p = 0.002)	CVD Death, MI, Stroke	0.64 [-0.24, 1.66]
FAST^(50) ^(N = 164)	Pravastatin	Significant Benefit (p < 0.001)	CVD Death, MI	0.32 [0.10, 1.06]
Pooled Estimate		-0.012 [-0.016, -0.007]**		0.48 [0.30, 0.78]

*Arms used in meta-analysis; **Excludes FAST

### Do IMT measurements meet clinical criteria for surrogate markers of cardiovascular disease events?

The three criteria described by Boissel, et al. [6] for surrogate markers relate to efficiency, linkage, and congruency and will be described in turn.

#### B1: Efficiency

Carotid IMT has been widely used in clinical trials. Reliable protocols have been established for its measurement and it is arguably more sensitive to the effects of interventions than cardiovascular disease events. Six of seven clinical trials in Table 1 reported a significant beneficial impact of statins on IMT progression with respect to their primary IMT outcome measure; the seventh trial, PLAC-II found no significant impact on its primary IMT outcome measure, but reported a significant impact on a secondary IMT measure. In six of seven trials, there were beneficial trends with respect to reported cardiovascular disease endpoints; however only for one trial (ACAPS) did this trend reach nominal statistical significance. Thus, while IMT measures were sufficiently sensitive so that benefit could be established within trials of this size, the general benefit with respect to cardiovascular events could not be generally established.

#### B2: Linkage

The strong association between carotid IMT and cardiovascular events has been demonstrated repeatedly. For example, the Cardiovascular Health Study, found it to be the risk factor most strongly associated with incident cardiovascular events [19]. In the Rotterdam Study, Del Sol, et al. found that a single carotid IMT measurement was of the same importance as a battery of commonly used risk factors in the prediction of CHD and CVD [20]. The Atherosclerosis Risk in Communities (ARIC) study found that carotid IMT of 1 mm or more was associated with two to five times the increased hazard of CHD and four to eight times the increased hazard of stroke [21,22]. Using a nested case-control approach and a mean duration of follow-up of 2.7 years, the Rotterdam Study found that per standard deviation increase (0.16 mm) in IMT, the odds ratio for stroke was 1.41 and for myocardial infarction was 1.43 [23].

Atherosclerosis is a manifestation of the pathophysiology underlying cardiovascular disease. The links between carotid IMT and atherosclerosis are well-established and IMT measures, as markers of atherosclerosis, have contributed greatly to the understanding of atherosclerosis progression [24,25]. These measures have characterized the role of many risk factors for atherosclerosis and currently serve the basis for several studies examining its genetics. The mechanisms by which atherosclerosis is causally related to cardiovascular events are also well-established.

#### B3: Congruency

IMT (continuous) and events (categorical) represent different measurement scales, thus it is difficult to argue they are influenced by statin therapy to quantitatively similar degrees. We drew evidence that the impacts are qualitatively similar using a meta-analysis of the clinical trials listed in Table 1 and developed pooled estimates of the relative impact of HMG-CoA reductase inhibitor (statin) therapy on IMT progression and on the odds ratio of cardiovascular endpoints [26]. Because standard errors for IMT changes were not reported for the FAST trial, it was excluded from this analysis. Across the trials, statin therapy was associated with an average decrease of IMT progression of 0.012 mm/yr with 95% confidence interval [-0.016, -0.007]. This pooled estimate confirms with greater precision the results from the individual trials. More importantly, the meta-analysis yields a significant odds ratio of 0.48 [0.30, 0.78] for the reduction cardiovascular events associated with statin therapy. Thus, a meta-analysis across a number of trials demonstrates a benefit with respect to cardiovascular disease events that is congruent with the benefits established by individual IMT trials.

### Do IMT measurements meet the criteria of statistical criteria for surrogate markers of cardiovascular disease events?

The four criteria of Prentice [7] are as follows.

#### P1: Impact of Interventions on Endpoint

There is convincing evidence, some of which is summarized in the meta-analysis described above, that statin therapy reduces the risk of cardiovascular events, to the extent that this is now an indication for their use.

#### P2: Impact of Interventions on Carotid IMT

As noted above, this association is supported by the results of our meta-analysis (Table 1) and elsewhere (e.g. [4]).

#### P3: Link Between Carotid IMT and Cardiovascular Events

The considerable evidence of this association has been discussed above.

#### P4: Conditional Independence Between Statin Therapy and Cardiovascular Events Given Carotid IMT

We know of no published literature that examines this conditional independence for statin therapies. Such a study is difficult to mount as it requires both sufficient power to demonstrate the relative impact on IMT progression of an intervention and sufficient size and follow-up time after this demonstration to assess the ability of measured IMT progression to account for subsequent risk. The only published account to examine the conditional independence of cardiovascular events given carotid IMT is for colestipol-niacin therapy in the Cholesterol Lowering Atherosclerosis Study (CLAS) clinical trial [27]. The 2-year CLAS trial demonstrated that colestipol-niacin therapy reduced IMT progression [28]; the trial cohort was surveyed an average of 8.8 years after the conclusion of CLAS to tally post-trial incidence of coronary events (nonfatal MI, coronary death, and coronary artery revascularization). These investigators found that while treatment assignment, by itself, was significantly related to occurrence of these events (relative risk 0.41; p = 0.01), when on-study IMT progression was included as a covariate, this relationship evaporated (relative risk 1.1; p > 0.2). Friedman, et al. use the term proportion of treatment effect captured (PTE) to describe how well a surrogate marker meets criterion P4 [29]; at face value, the findings from CLAS produce an estimate that PTE exceeds 1.

In our meta-analysis, when IMT progression is included as a covariate in regression models linking cardiovascular disease events to statin treatment, the relative odds ratio is mediated from 0.48 (as tabulated below) to 0.64 and is no longer statistically significant (p = 0.13). This suggests that changes in IMT may account for some, but not all, of the effect of statins on cardiovascular events (i.e. a PTE of 0.3). Several issues complicate this argument, however. Even if a surrogate successfully meets Prentice's criteria for surrogacy within individual trials, because designs, cohorts, and endpoints vary it is to be expected that a surrogate would only account for some, not all, of treatment effects in regression models across trials. Secondly, like many markers, IMT is subject to measurement error and that is not insubstantial. This measurement error, if uncorrected, may lead to marked underestimates of relationships [30]: measured IMT progression may appear to account for less of the relationship between interventions and events than true progression. These issues obscure the validation of surrogacy from meta-analyses based on published summary statistics. We can only conclude that IMT progression may account for at least some of the treatment effects attributable to statin therapy, but that it is difficult to quantitate the degree of this relationship and that full surrogacy cannot be ruled out.

## Summary

We have examined, in a structured and rigorous manner, the evidence that carotid IMT progression may serve as a surrogate for cardiovascular disease endpoints in statin trials. Each of the criteria for surrogacy described by Boissel, et al. appears to be met. The first three of Prentice's criteria are met, and the fourth is met by the one published study for which it can be evaluated (although not for statin therapy). Meta-analyses of statin trials provide support for Boissel's criteria and the first three of Prentice's criteria, and are not inconsistent for Prentice's fourth criterion.

It is possible that these arguments may generalize to other agents whose mechanisms are similar to statins, however additional analyses, based on criteria for surrogate outcomes, would be required to make this extension.

## Competing interests

MAE received an honorarium from Sankyo Pharma, Inc for a meeting during which ideas for this manuscript were developed. He is an occasional consultant to other companies concerning the design of clinical trials involving carotid ultrasonography. DHO serves on data safety and monitoring boards for Pfizer and Astra/Zeneca and serves as consultant to Sankyo Pharma and to Merck. JGT received an honorarium from Sankyo Pharma, Inc for a portion of this work. TO has no competing interests. GE has received an honorarium and consulting fees from AstraZeneca Pharmaceuticals for assistance in the planning and implementation ofa clinical trial involving carotid ultrasonography and statin therapy. He also serves as an occasional consultant to other companies on the design and conduct of trials involving carotid ultrasonography in which statins may be included as background therapies, but are not part of the experimental intervention. HM is an occasional consultant to Sankyo Pharma, including attending the meeting during which ideas for this manuscript were developed. He very occasionally consults with other Pharma companies like MSD, Essex, and Lilly. He is a consultant to Boston Scientific for interventional cardiology and intravascular ultrasound and is also a regular teacher in carotid stenting for the Guidant and Cordis companies.

## Authors' contributions

MAE, DHO, and HM conceived and drafted this manuscript. MAE, JGT, and TM organized and conducted its meta-analysis. GE provided oversight to analyses and contributed to interpretation of results. All authors read and approved the final manuscript.
